# Exploring the Anticancer Effect of *Artemisia herba‐alba* on Colorectal Cancer: Insights From Eight Colorectal Cancer Cell Lines

**DOI:** 10.1002/fsn3.4715

**Published:** 2024-12-31

**Authors:** Lara J. Bou Malhab, Amani A. Harb, Leen Eldohaji, Jalal Taneera, Hamza M. Al‐Hroub, Ahmad Abuhelwa, Karem H. Alzoubi, Bashaer Abu‐Irmaileh, Mohammad Hudaib, Jehad Almaliti, Wael M. Abdel‐Rahman, Abdallah Shanableh, Mohammad H. Semreen, Waseem El‐Huneidi, Eman Abu‐Gharbieh, Yasser Bustanji

**Affiliations:** ^1^ Sharjah Institute for Medical Research University of Sharjah Sharjah UAE; ^2^ Department of Basic Sciences, Faculty of Arts and Sciences Al‐Ahliyya Amman University Amman Jordan; ^3^ College of Medicine University of Sharjah Sharjah UAE; ^4^ College of Pharmacy University of Sharjah Sharjah UAE; ^5^ Hamdi Mango Center for Scientific Research The University of Jordan Amman Jordan; ^6^ School of Pharmacy The University of Jordan Amman Jordan; ^7^ Skaggs School of Pharmacy and Pharmaceutical Sciences University of California San Diego San Diego California USA; ^8^ Department of Medical Laboratory Sciences, College of Health Sciences University of Sharjah Sharjah UAE; ^9^ Research Institute of Science and Engineering (RISE) University of Sharjah Sharjah UAE

**Keywords:** apoptosis, *Artemisia herba‐alba*, cell cycle arrest, colorectal cancer, cytotoxicity, GC–MS analysis, PI3K/AKT/mTOR pathway

## Abstract

Colorectal cancer (CRC) is a prevalent and deadly disease, necessitating the exploration of novel therapeutic strategies. Traditional chemotherapy often encounters drug resistance and adverse side effects, highlighting the need for alternative approaches. *Artemisia herba‐alba* , a plant rich in phytochemical constituents, was investigated for its potential as an anticancer agent against colorectal cancer (CRC). The primary objective of this study was to investigate the cytotoxic effects of the methanolic extract of *A. herba‐alba* on eight CRC cell lines including: Caco‐2, DLD1, RKO^+/+^p53, RKO^−/−^p53, HCT^+/+^p53, HCT^−/−^p53, SW620, and SW480. Specifically, the study investigated the extract's impact on cell viability, apoptosis, cell cycle progression, and effects on the PI3K/AKT/mTOR signaling pathway. Chemical derivatization and Gas Chromatography–Mass Spectrometry (GC–MS) analysis revealed a diverse array of bioactive compounds, including ephedrine, hydroxyflavone, quinolinic acid, 4‐hydroxybenzoic acid, borneol, β‐eudesmol, and camphor, known for their cytotoxic properties. The methanolic extract of *A. herba‐alba* exhibited varying degrees of cytotoxicity across a panel of CRC cell lines, with IC_50_ values indicating differential sensitivity. The extract triggered apoptosis in many cell lines, irrespective of p53 status. Importantly, *A. herba‐alba* extract caused G2‐M phase cell cycle arrest in CRC cells, accompanied by a decrease in Cyclin B1 and CDK1 expression. Furthermore, the extract demonstrated an inhibitory effect on the PI3K/AKT/mTOR pathway, crucial in cancer progression. These findings highlight the promising anticancer potential of *Artemisia herba‐alba* as a valuable resource for innovative CRC treatments. Further research is warranted to elucidate its specific anticancer characteristics and explore its potential incorporation into future cancer therapy approaches.

## Graphical abstract

The methanolic extract of *Artemisia herba‐alba* demonstrates anticancer properties against colorectal cancer (CRC) by inhibiting cell viability, inducing apoptosis, and arresting the cell cycle in the G2‐M phase. It affects the PI3K/AKT/mTOR signaling pathway and exhibits cytotoxic effects across various CRC cell lines, with differing sensitivity. Chemical analysis identified bioactive compounds like ephedrine, hydroxyflavone, and camphor, contributing to its therapeutic potential. These findings suggest *A. herba‐alba* could be a valuable resource for developing novel CRC treatments.

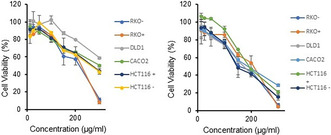

## 1 Introduction

Colorectal cancer (CRC) is a significant global health issue and is among the leading causes of cancer‐related mortality worldwide. There has been a steady increase in the occurrence of CRC, and future estimates suggest a notable surge in both the number of cases and mortality rates in the upcoming years (Murphy and Zaki 2024). CRC cancer is defined as the uncontrolled growth and spread of abnormal cells in the colon or rectum. It frequently metastasizes to the liver, lungs, and other parts of the gastrointestinal system, which makes treatment and prognosis more challenging (Marabotto et al. 2022). The difficulty in treating CRC arises from the adverse effects associated with conventional therapeutic approaches. Chemotherapeutic agents have played a crucial role in the treatment of CRC. Nevertheless, the use of synthetic chemical anticancer drugs can result undesirable outcomes, such as drug resistance and various adverse effects like immunosuppression, anemia, and organ toxicity. Given the challenges associated with chemotherapy resistance and cytotoxicity, it is essential to explore alternative therapeutic methods that offer improved efficacy and fewer adverse effects (Kasabri et al. 2014; Selek et al. 2018; Gmeiner 2024).

Natural compounds and plant extracts have become a remarkable source of medicinal agents for managing various chronic diseases, presenting a promising opportunity for advancing innovative anticancer treatments (Bajes, Oran, and Bustanji 2021; Al‐Eisawi et al. 2022; Wang et al. 2022; Bayrakçeken Güven et al. 2023; Memarzia et al. 2023; Mia et al. 2023; Sirajudeen et al. 2024). Plants contain various primary and secondary metabolites, such as alkaloids, flavonoids, and phenolic compounds. These compounds have been linked to various pharmacological properties, including antioxidant, antiproliferative, anti‐inflammatory, antimicrobial, antidiabetic, and chemoprotective effects (Mohammad et al. 2010; Bustanji et al. 2011, 2023; Kasabri et al. 2017; Harb, Bustanji, and Abdalla 2018; Abu‐Gharbieh et al. 2019; Paul et al. 2024; Witayateeraporn, Hardianti, and Pongrakhananon 2024).

*Artemisia herba‐alba* is a perennial dwarf shrub that belongs to the Asteraceae family. It is characterized by its greenish‐silver color and is well‐suited to dry and semi‐arid areas, especially in the Mediterranean (Yener et al. 2020; Dmour et al. 2024).

In traditional medicine, *A. herba‐alba* has been utilized to address various health issues, including diabetes, hair loss, fever, diarrhea, vomiting, bronchitis, gastrointestinal disturbances, and muscular pains (Elbahaie et al. 2023). The unique chemical composition of this plant is responsible for its potent antibacterial, antispasmodic, antifungal, and vasorelaxant properties (Mohamed et al. 2010; Souhila, Fatma Zohra, and Tahar 2019). It is worth mentioning that this plant demonstrates significant polymorphism in its composition. This variability has led to the identification of different chemotypes worldwide, which has significantly attracted the scientific community (Benchohra, Ahmed, and Merah 2023; Elwardani et al. 2024).

The phytochemical composition of *A. herba‐alba* is diverse, including flavonoids, phenolics, alkaloids, monoterpenoids, sesquiterpenes, pigments, steroids, and essential oils (Mohamed et al. 2010; Souhila, Fatma Zohra, and Tahar 2019). These compounds have demonstrated the potential to boost the immune system and offer a broad spectrum of possible applications, including antimicrobial, antioxidant, and anti‐inflammatory properties. Additionally, many of these identified compounds exhibit a wide array of bioactive chemicals, extensively documented for their cytotoxic effects (Mrabti et al. 2023; El Hajli et al. 2024). The plants' extracts have exhibited potential in anticancer research, displaying cytotoxic effects on a range of human cancer cell lines, such as colon cancer, laryngeal carcinoma, bladder carcinoma, and myelogenous leukemia (Ahmed et al. 2020; Ouahdani et al. 2021; Hasan et al. 2022; Al‐Qbilat and Atrooz 2024).

The current study underscores the extract's selective cytotoxicity against CRC cell lines, highlighting its potential to serve as a complementary treatment to existing cancer treatment with more efficacious and less harmful alternatives. Our aim is to elucidate the anticancer potential of *A. herba‐alba* by investigating its effects on cell survival, apoptotic mechanisms, cell cycle control, and regulation of the PI3K/AKT/mTOR pathway.

## 2 Materials and Methods

### 2.1 Plant Material

*Artemisia herba‐alba* aerial parts were gathered from southern Jordan in May 2021. Prof. M Hudaib, an esteemed expert in Pharmacognosy and Phytochemistry at the University of Jordan's School of Pharmacy, conducted the botanical identification of these specimens. A voucher specimen labeled as Art‐2022‐5‐07 has been safely stored at the School of Pharmacy's repository at the University of Jordan. After collection, the aerial parts were air‐dried at room temperature, and protected from direct sunlight to avoid deterioration of sensitive components. After drying, the plant material was mechanically ground into a fine powder with particles no larger than 0.5 mm (Bajes, Oran, and Bustanji 2022, 2023). This preliminary step was done to enhance the effectiveness of the next extraction process, guaranteeing the highest possible amount of the extracted phytochemicals.

### 2.2 Extraction of Plant Material

The extraction of *A. herba‐alba* with methanol was carried out according to the following procedure: 100 g of the plant's dried and powdered aerial parts were initially mixed with 1000 mL methanol (99.7% purity). The mixture was allowed to soak at room temperature for 72 h, protected from light by covering the container with aluminum foil. The mixture was continuously agitated by continuous stirring to ensure thorough mixing and extraction. After the maceration period, the mixture was filtered with Whatman filter paper (No. 1) to separate the liquid extract from the plant residue. The methanol extract was concentrated by evaporation using a rotary evaporator. The system was configured to function at a decreased pressure of 12 mbar and a water bath temperature of 50°C. A rotating speed setting of 3 rounds/min was used to remove methanol until the extract was dried entirely (Yousef et al. 2021; Althaher, Oran, and Bustanji 2022). The concentrated extract was stored in 50 mL Falcon tubes, covered with aluminum foil to shield it from light, and kept at a temperature of 4°C for preservation.

### 2.3 Phytochemical Analysis

The chemical profiling of the methanolic extract of *A. herba‐alba* was conducted using GC–MS derivatization analysis. In the sample preparation phase, 80 μL of *N*‐Methyl‐*N*‐(trimethylsilyl) trifluoroacetamide with 1% trimethylchlorosilane (MSTFA + 1% TMCS) was added to approximately 10 mg of the plant extract. This mixture was then incubated at 60°C for 30 min. Following this, 100 μL of *n*‐hexane was added, and the mixture underwent a second incubation at the same temperature for 15 min. After these incubation steps, a 1 μL sample was carefully injected into the GC–MS for analysis (Dawra et al. 2024).

The analysis was conducted on a GC–MS system, QP2010 Ultra model from Shimadzu Corp., Japan. This system was equipped with an SLB‐5ms fused silica capillary column, measuring 30 m in length and 0.25 mm in diameter, with a film thickness of 0.25 μm. The sample injection was performed in a splitless mode using an AOC‐20i auto‐sampler, and the volume of the sample injected was precisely 1.0 μL. High‐purity helium gas was used as the carrier gas with a purity level of 99.999%. It was maintained at a controlled pressure of 90 kPa and the flow rate through the column was set at 1.32 mL/min.

The GC parameters were set with an injection temperature of 270.00°C and the sampling time was fixed at 1 min. The oven temperature program started at 60.0°C, holding this temperature for 3 min. Then, the temperature was increased at a rate of 7.0°C per minute up to 140.0°C, followed by a further increase at a rate of 5.0°C per minute until it reached 300.0°C, where it was held for an additional 5 min. The total run time for the GC was 51.43 min. For the MS parameters, the ion source temperature was set at 200.00°C and the interface temperature at 270.00°C. The mass spectrometer operated in a scan mode, covering a range from 50 to 650 m/z. This detailed procedure ensured accurate preparation and analysis for the GC–MS chemical profiling of the methanolic extract of *A. herba‐alba* .

### 2.4 Cell Lines

Cells for human colorectal cancer, including Caco‐2, DLD1, RKO^+/+^p53, RKO^−/−^p53, HCT^+/+^p53, HCT^−/−^p53, SW620, and SW480, were cultured with either Dulbecco's Modified Eagle's Medium (DMEMD) or Roswell Park Memorial Institute Medium (RPMI 1640) supplemented with 100 U/mL streptomycin, 100 U/mL penicillin, and 10% fetal bovine serum (FBS). Cells were cultured in 5% carbon dioxide (CO_2_) at 37°C. Cells were subcultured every 72 h and the cells were transferred into containers when they reached almost 80% confluency.

### 2.5 Cytotoxicity Assay

MTT assay is widely used to determine the functional state of the mitochondria, where live cells reduce yellow tetrazolium MTT salt to blue MTT formazan with the mitochondrial dehydrogenase enzyme indicating cell viability (Althaher, Oran, and Bustanji 2020, 2021; Bou Malhab et al. 2023). A 20 mg of the plant's extract was dissolved in 1 mL of DMSO to create a stock solution. Concentrations between 12.5 and 300 μg/mL (0, 12.5, 25, 50, 100, 150, 200, 300 μg/mL) were prepared from this solution and applied to the cells for 24 and 48 h in triplicates after they had been seeded into a 96‐well plate with 5 × 10^3^ cells per well. After treatment, MTT (3‐[4,5‐dimethylthiazol‐2‐yl] 2,5 diphenyl tetrazolium bromide) [5 mg/mL in phosphate‐buffered saline (PBS)] was added to the medium and the cells were incubated at 37°C between 2 and 4 h to allow for the reduction of MTT. DMSO (100 μL) was added to dissolve MTT crystals and then incubated for 5 min. The absorbance at 570 nm was measured using a microtiter plate reader, and the absorbance of the treated cultures was contrasted with that of the control cultures that were left untreated to calculate the rate of cell proliferation. Cisplatin was used as positive control. Vehicle (DMSO) as control experiments were also performed (Abu‐Rish et al. 2016; Alhourani et al. 2020).

### 2.6 Apoptosis Assay (Annexin V/PI)

Cells were seeded in 6‐well plates at 60% confluency and then treated with *A. herba‐alba* extract at a concentration equal to the IC_50_ value for 48 h. After treatment, cells were rinsed twice with warm PBS and collected. The collected cells were subsequently treated with Annexin V/PI staining buffer for 10 min. Following the staining process, cells were rinsed and then stained with Propidium Iodide (PI) for 15 min, following the manufacturer's instructions (Yousef et al. 2021; Althaher, Oran, and Bustanji 2022; Bou Malhab et al. 2023). Cells were examined for apoptosis and necrosis using a flow cytometer (BD FACS Aria III; Becton Dickinson) with excitation at 488 nm. Fluorescence detection was performed at two specific wavelengths: 530/30 nm for Annexin V and 615 nm for PI (adjusted from 502 nm to match conventional PI detection wavelengths). Early apoptotic cells were identified based on Annexin V positive, late apoptotic cells were identified by dual positivity for Annexin V and PI, and necrotic cells were identified by exclusive PI positivity.

Data analysis was performed utilizing Watson's pragmatic algorithm and the FlowJo program to produce relevant figures and analyze data. This analysis divided the cells into four different quadrants depending on their apoptotic/non‐apoptotic status. The percentage of live cells will be represented in quadrant 4 (Q4), whereas early apoptotic cells will be represented in Q2 through annexin V and phosphatidyl serine interaction. Propidium iodide (PI) will recognize late apoptotic (Q3) and necrotic cells (Q1) through its passage into the nucleus due to cell membrane damage and its ability to bind to DNA double helix (Bou Malhab et al. 2023).

### 2.7 Cell Cycle Analysis

Colorectal cell lines were cultured separately in six‐well plates to 60% confluency before treatment with *Artemisia herba‐alba* extract for 48 h. Post‐treatment with their respective IC_50_ concentration, cells were fixed in 70% ethanol at −20°C overnight, washed, and then stained with Propidium Iodide (PI) and DNAse‐free RNase for 30 min. The cell cycle phase progression was then analyzed using a flow cytometry platform (Bou Malhab et al. 2023).

### 2.8 Western Blot

Cells were seeded in six well plates at 60% confluency and treated with *A. herba‐alba* for 48 h. Cells were then washed twice with PBS and lysed with RIPA buffer containing protease inhibitor cocktail (Sigma‐Aldrich) (Bou Malhab et al. 2023; Tarawneh et al. 2023). Protein concentration was determined by using Bradford method from Bio‐Rad. 12% SDS‐PAGE then separated thirty micrograms of protein, transferred to a nitrocellulose membrane from Bio‐Rad, and blocked for 1 h with 5% BSA (Sigma‐Aldrich). The membrane was washed with Tris‐buffered saline with 0.1% Tween 20 and incubated overnight at 4°C with primary antibodies against CyclinB1, CDK1, and actin (Cell Signaling Technology). The membrane was then treated for an hour with 1:1000 dilution of the secondary antibodies anti‐rabbit or anti‐mouse purchased from Cell Signaling Technology. Chemiluminescence from Thermo Fisher Scientific was used to see the bands, and the band density was calculated using Bio‐Rad's Image Lab software. Actin was used as a reference standard for normalization.

## 3 Results

### 3.1 GC–MS Analysis

Gas Chromatography–Mass Spectrometry (GC–MS) examination of *A. herba‐alba* revealed the presence of a wide variety of phytochemical constituents. The chemical composition of *A. herba‐alba* is outlined in Table S1. Compounds' identification was established based on their retention times and mass spectra (MS) matched with electronic MS‐libraries and external standards. The identified principles with their retention times and percentages of peak area (%) are outlined in Figure S1 and Table S1. The identified groups include amino acids, aromatic compounds, fatty acids, flavonoid glycosides, alkanes, heterocyclic compounds, organic acids, organic alcohols, phenolic compounds, sterols, sugars, and their derivatives, as well as mono‐ and sesquiterpenes. Various compounds with proven cytotoxic properties were identified, such as ephedrine (an alkaloid), hydroxyflavone (a flavonoid), quinolinic acid (an organic acid), 4‐hydroxybenzoic acid (a phenolic compound), borneol (a monoterpene alcohol), β‐eudesmol (a sesquiterpene alcohol), and camphor (a monoterpene ketone) (Boğa et al. 2016; Chen et al. 2016; Seidel et al. 2016; Hernandez‐Martinez et al. 2017; Biswas et al. 2018; Ceylan et al. 2021; Li et al. 2021, 2023, 2024; Myint et al. 2021; Altay et al. 2022; Ullah et al. 2022; Basson et al. 2023; Ogunlakin et al. 2023; Singh et al. 2023; Lin et al. 2024).

### 3.2 Effect of *A. herba‐alba* on Colorectal Cell Viability

**FIGURE 1:**
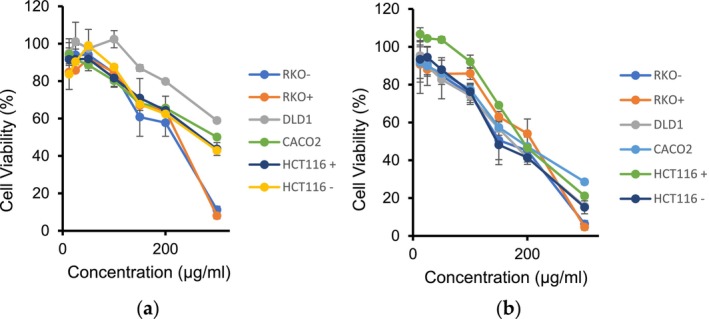
Inhibitory effect escalating concentration of *A. herba‐alba* extract on cell viability of various colon cancer cell lines after (A) 24 h and (B) 48 h of incubation.

The cytotoxic effect of the methanolic extract of *Artemisia herba‐alba* was evaluated across a panel of colon cancer cell lines, including Caco2, DLD1, SW620, HCT116^+/+^p53, HCT116−/−p53, RKO^+/+^p53, and RKO^−/−^p53, SW620, and SW480. The IC_50_ values, representing the concentration of extract required to inhibit 50% of cell viability, were calculated using regression analysis of cell viability percentages at varying concentrations of the extracts, as shown in Table 1 and Figure 1.

**TABLE 1:** The cytotoxic effect of methanolic extract of *Artemisia herba‐alba* on colon cancer cell lines, 24 and 48 h after exposure.

Cell line (48 h)	IC_50_ (μg/mL) ± SEM (24 h)	IC_50_ (μg/mL) ± SEM (48 h)
Caco2	256.14 ± 24.98	182.78 ± 16.33
DLD1	463.17 ± 175.80	177.76 ± 3.02
HCT116^+/+^p53	265.99 ± 1.38	205.57 ± 1.84
HCT116^−/−^p53	206.63 ± 42.94	187.97 ± 15.76
RKO^−/−^p53	197.36 ± 1.84	170.54 ± 5.28
RKO^+/+^p53	250.68 ± 59.30	182.47 ± 1.27
SW620	Not active	Not active
SW480	Not active	Not active

Table 1 and Figure 1 present the cytotoxic responses of various colon cancer cell lines to the methanolic extract of *Artemisia herba‐alba* . The results reveal a range of sensitivities to the extract. After 48 h of incubation, a decrease in IC_50_ values was observed in CRC cell lines, indicating increased cytotoxicity with prolonged exposure.

The Caco2 cell line had an IC_50_ of 182.78 ± 16.33 μg/mL, and the DLD1 cell line had a slightly lower IC_50_ of 177.76 ± 3.02 μg/mL, indicating similar sensitivity levels between the two cell lines. In contrast, the SW620 and SW480 cell lines showed no activity, suggesting resistance to the extract's cytotoxic properties under the given conditions.

The HCT116 cell lines, both the p53 variant (IC_50_: 205.57 ± 1.84 μg/mL) and the non‐p53 variant (IC_50_: 187.97 ± 15.76 μg/mL), exhibited slight differences in responsiveness, possibly due to the p53 status. Similarly, the RKO cell lines, regardless of p53 expression, displayed IC_50_ values of 170.54 ± 5.28 and 182.471.27 μg/mL, respectively, indicating a minor difference in their response to the extract.

### 3.3 *A. herba alba* Promotes Cell Death in CRC Cells

Cellular homeostasis relies heavily on the controlled mechanism of cell death (apoptosis) to remove non‐functional cells. Therefore, targeting apoptotic pathways has become a viable strategy for creating anticancer treatments.

**FIGURE 2:**
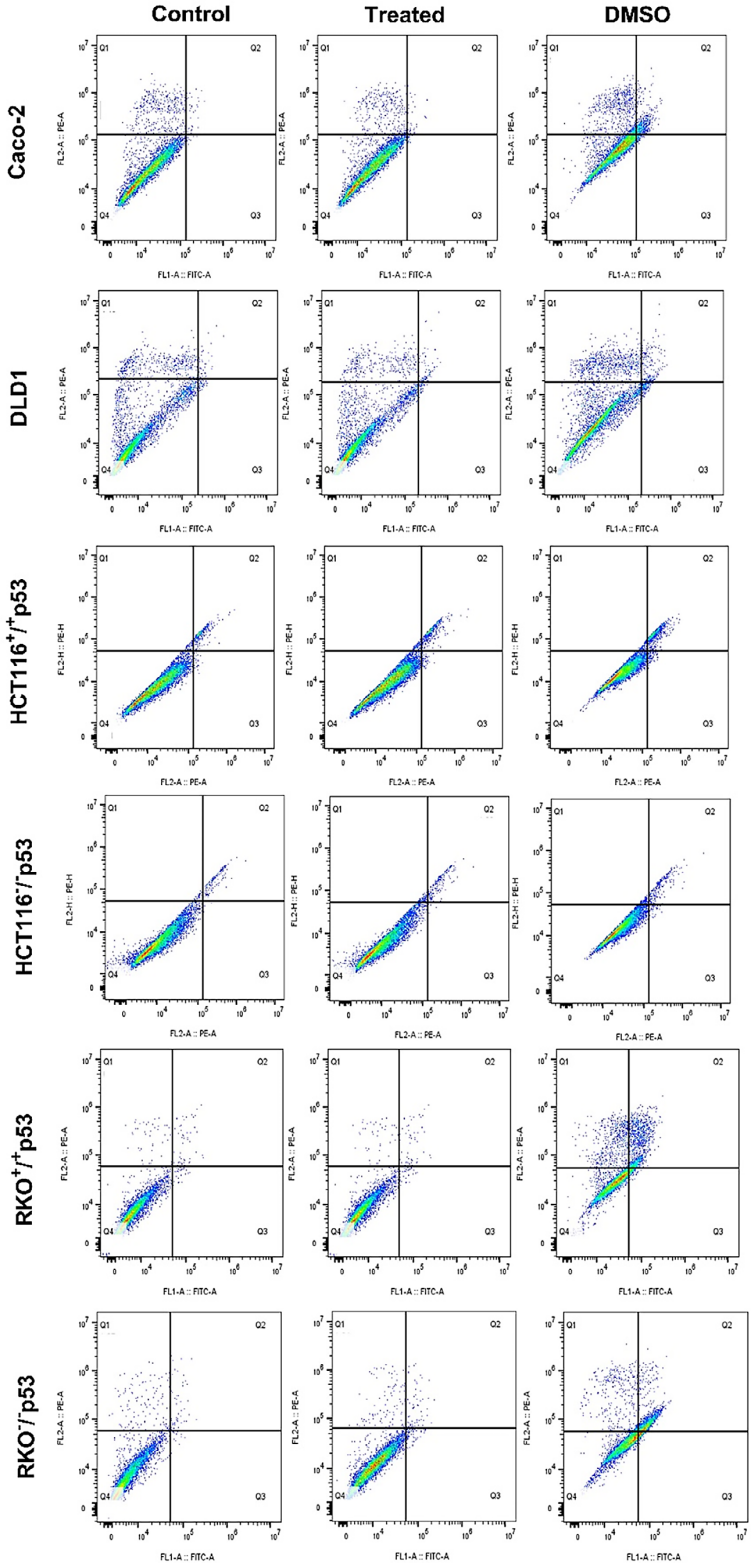
*A. herba‐alba* promotes cell death in all CRC cell lines tested. The percentage of apoptotic and necrotic cells was assessed by flow cytometry using Annexin V/PI staining. Control cells were cultured in media, whereas treated cells were treated with the extract for 48 h with their corresponding IC50 concentration. DMSO was used as a negative control. Q1, Q2, Q3, and Q4 represent the average of three independent experiments is represented.

In this study, a significant increase in cell mortality was observed in different colorectal cancer cell lines after treatment, with differing degrees of escalation across the cells (Figure 2; Table 2). Caco‐2 cells showed a rise in early and late apoptosis to 4.76% and 9.61%, respectively, from a 0.2% and 0.4% baseline in the untreated group. DLD1 cells showed increased apoptosis (early and late stages) and necrosis in response to herba‐alba, accounting for 4.58% and 5.7% of the population, respectively. The RKO isogenic cell lines, each with different p53 statuses, likewise showed a significant apoptotic response to the therapy. Apoptosis was observed in 12.5% of the p53 competent RKO cells, a significant rise from less than 1% in the untreated group, and a notable 32.82% in the p53 deficient RKO cells, compared to their untreated counterparts. The HCT116 isogenic cells responded differently to the extract, with the cells containing functional p53 displaying a higher reaction to treatment compared to the cells without p53. The cell death rate post‐treatment was 8.74% in p53 competent cells and less than 1% in untreated cells.

**TABLE 2:** The percentage of apoptotic and necrotic cells, with the average values derived from three independent experiments ±SD.

Annexin FITC/PI	Necrosis	Early apoptosis	Late apoptosis	Live cells
	Q1	Q2	Q3	Q4
Caco2				
Control	2.5 ± 1.27	0.24 ± 0.02	0.4 ± 0.01	96.85 ± 0.62
Treated	4.9 ± 3.21	4.76 ± 2.33	9.61 ± 1.83	80.8 ± 0.92
DMSO	2.0 ± 0.94	0.38 ± 0.09	0.575 ± 0.11	96.95 ± 0.74
DLD1				
Control	1.0 ± 0.19	0.27 ± 0.16	0.355 ± 0.06	96.4 ± 0.03
Treated	5.7 ± 0.79	2.12 ± 0.26	2.46 ± 0.42	89.65 ± 0.05
DMSO	2.9 ± 0.12	0.55 ± 0.035	0.68 ± 0.13	95.8 ± 0.28
RKO^+/+^p53				
Control	0.44 ± 0.003	0.44 ± 0.01	0.53 ± 0.08	98.5 ± 0.07
Treated	5.5 ± 0.24	3.44 ± 2.27	9.19 ± 3.97	86.4 ± 3.67
DMSO	0.40 ± 0.003	0.41 ± 0.07	0.66 ± 0.07	98.5 ± 0.10
RKO^−/−^p53				
Control	0.98 ± 0.10	0.14 ± 0.01	0.41 ± 0.12	98 ± 0.10
Treated	1.0 ± 1.06	7.62 ± 1.03	25.2 ± 0.85	63.5 ± 1.27
DMSO	0.75 ± 0.20	0.50 ± 0.24	0.49 ± 0.14	98.25 ± 0.60
HCT116^+/+^p53				
Control	0.54 ± 0.01	0.2 ± 0.04	0.1 ± 0.01	99.1 ± 0.14
Treated	3.19 ± 1.43	1.03 ± 0.23	4.52 ± 2.70	91.55 ± 1.73
DMSO	0.78 ± 0.13	0.53 ± 0.11	2.57 ± 1.79	96.1 ± 2.05
HCT116^−/−^p53				
Control	0.45 ± 0.02	0.10 ± 0.02	0.24 ± 0.14	99.1 ± 0.14
Treated	2.2 ± 0.22	0.53 ± 0.01	2.2 ± 0.15	95.7 ± 0.24
DMSO	0.62 ± 0.13	0.24 ± 0.14	0.94 ± 0.63	98.2 ± 0.64

*Note:* The data is categorized into Q1 (representing necrosis), Q2 (late apoptosis), Q3 (early apoptosis), and Q4 (viable cells), each reflecting distinct stages of cellular status as determined through flow cytometric analysis.

### 3.4 *A. herba‐alba* Promotes G2‐M Cell Cycle Arrest in CRC Cells

**FIGURE 3:**
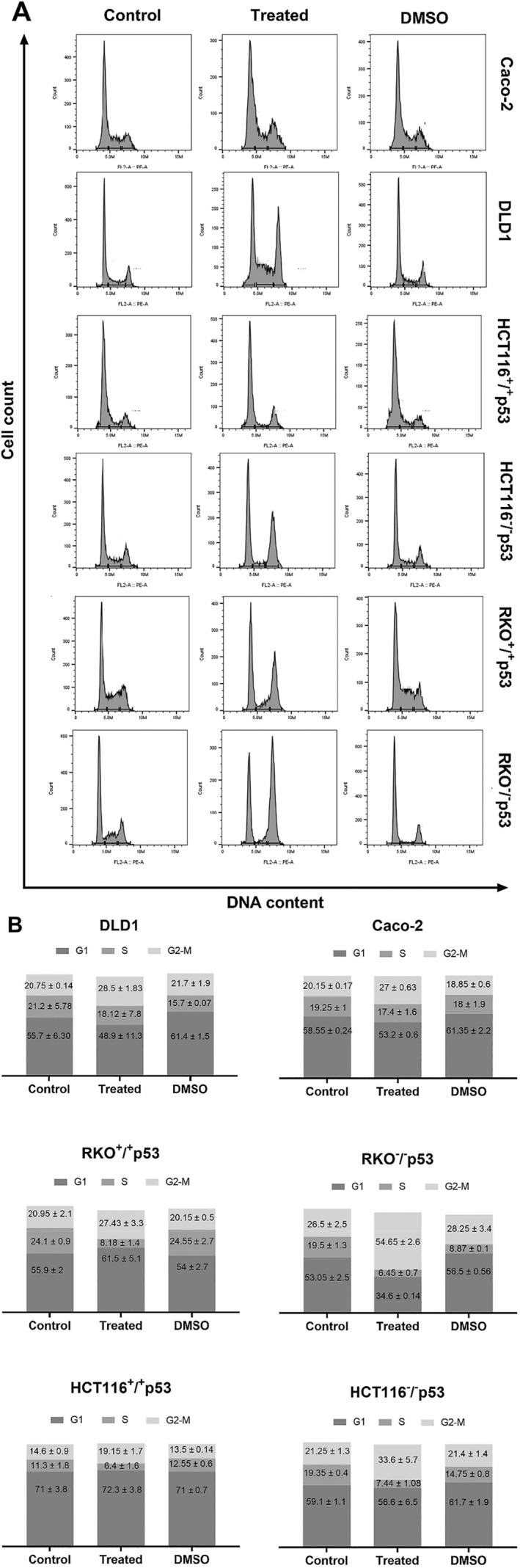
Cell cycle analysis in CRC lines after treatment with *A. herba‐alba*. (A) Treatment with *A. herba‐alba* leads to G2‐M phase accumulation in all CRC cell lines. (B) The average values of three independent experiments are presented as percentages of cells in G1, S, and G2‐M.

The cell cycle is a crucial biological process that regulates several cellular functions such as growth, DNA replication, and division. It is highly regulated process by phase‐specific proteins, particularly cyclins (Kalsbeek and Golsteyn 2017). These proteins form complexes with cyclin‐dependent kinases (CDKs) to initiate their activation, resulting in the phosphorylation of specific proteins and facilitating smooth transitions between the various phases of the cell cycle, namely G1, S, G2, and *M. cancer* cells are known to have an uncontrolled proliferation as a result of the aberrant activity of various cell cycle proteins, which has made targeting these proteins as an attractive target for cancer therapy (Kalsbeek and Golsteyn 2017). The present study examined the effects of *A. herba‐alba* on the investigated CRC cell lines and observed a significant arrest in the G2‐M phase after treatment with the plant extract. The DLD1, Caco‐2, RKO^+/+^p53, and HCT^+/+^p53 cell lines displayed a notable rise of approximately 10% in cells during the G2‐M phase. In contrast, the RKO^−/−^p53 and HCT^−/−^p53 lines exhibited more significant increases of approximately 30% and 13%, respectively (Figure 3A,B).

**FIGURE 4:**
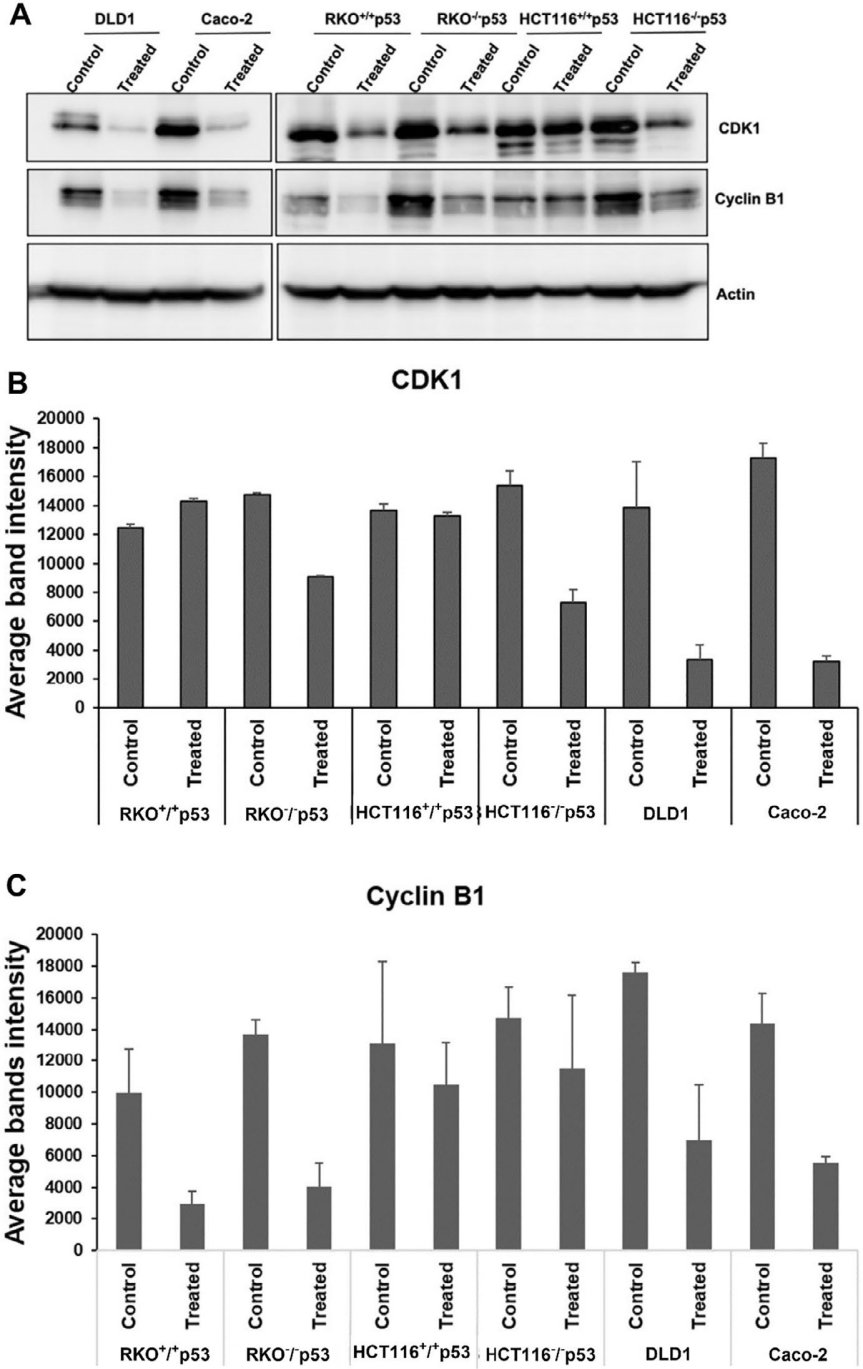
The effect of *A. herba‐alba* extract on different cell cycle regulatory and apoptotic markers proteins. (A) All CRC cell lines were treated with the extract for 48 h, harvested and monitored for the different proteins expression CyclinB1, CDK1. Actin was used as a loading control. (B) The quantitative analysis of cyclin B1 (C) for CDK band density after normalization to the loading control is represented.

The two major key regulators of the G2‐M phase are CyclinB1 and CDK1. The effect of *A. herba‐alba* treatment on the expressions of CyclinB1 and CDK1 was investigated by Western Blot analysis, as these proteins play a crucial role in regulating the G2‐M transition. The frevealed a decrease in the levels of Cyclin B1 and CDK1 expression in all CRC cell lines that were treated, as compared to the untreated cells. This highlights the extract's ability to regulate the course of the cell cycle and suggests a promising approach for cancer treatment (Figure 4).

### 3.5 *A. herba‐alba* Inhibits the PI3K/AKT/mTOR Pathway

**FIGURE 5:**
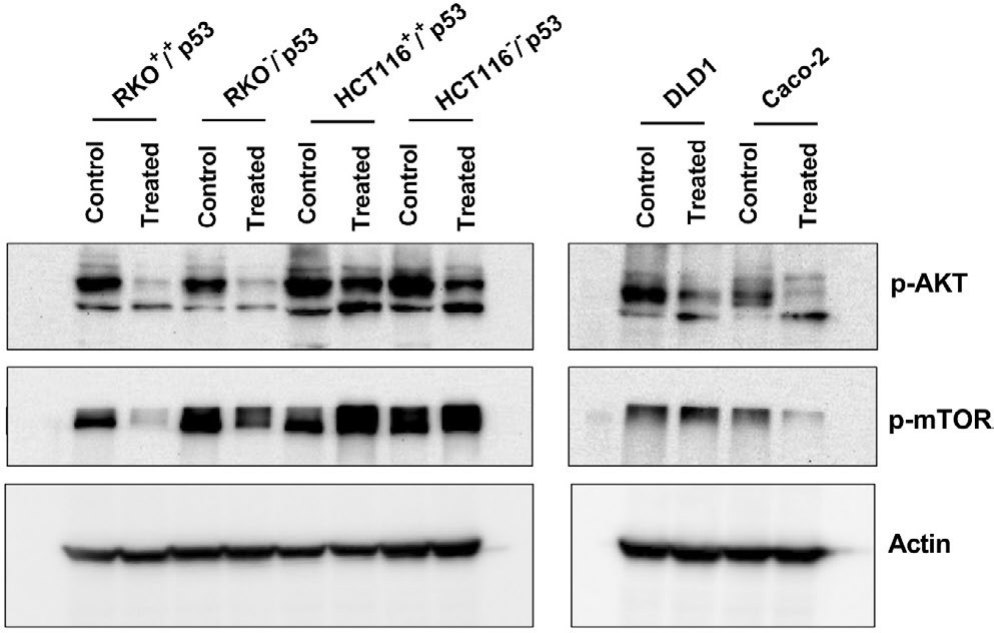
The effect of *A. herba‐alba* on different cell death markers p‐AKT and p‐mTOR.

The PI3K/AKT/mTOR is a classical signaling pathway involved in different biological processes such as autophagy, cell cycle progression, cell proliferation, metastasis, and apoptosis (Tarawneh et al. 2023). Nevertheless, this pathway is a common activated pathway in tumors, and Its overexpression has been reported in colorectal cancer. To understand the effect of *A. herba‐alba* on the PI3K/AKT/mTOR pathway, we treated all CRC cell lines with their respective IC_50_ for 48 h and we checked the expression of phosphorylated AKT and mTOR (Figure 5). Compared to the untreated conditions, all CRC cells showed less phosphorylation in either AKT and/or mTOR protein. RKO^+/+^p53, RKO^−/−^p53, and Caco‐2 showed a significant decrease in both AKT and mTOR phosphorylation, whereas HCT^−/−^p53 and DLD1 only AKT phosphorylation was affected.

## 4 Discussion

Historically, traditional plant‐based treatments have been highly esteemed for their therapeutic attributes in various societies, acting as a valuable source of bioactive substances. Scientific studies on these natural compounds have shown their ability to regulate different biological pathways involved in the pathogenesis of chronic diseases (Bajes, Almasri, and Bustanji 2020; Sayed et al. 2021; Fang et al. 2023; Rakotondrabe et al. 2023).

Our investigation showed through GC–MS analysis that the *A. herba‐alba* extract contains a broad spectrum of bioactive compounds with known cytotoxic activity, including ephedrine, hydroxyflavone, quinolinic acid, 4‐hydroxybenzoic acid, borneol, β‐eudesmol, and camphor, each contributing to its significant therapeutic potential in cancer treatment and management (Table 1). Ephedrine showed efficacy against breast cancer cells (Chen et al. 2016), while hydroxyflavone and its derivatives exhibited promising inhibitory actions against colorectal cancer, alongside providing relief from chemotherapy‐induced neuropathic pain (Biswas et al. 2018; Ullah et al. 2022; Ogunlakin et al. 2023). Quinolinic acid was identified as a potential agent for neurodegenerative disease management through neuritogenesis induction and its significant melanoma cytotoxicity (Hernandez‐Martinez et al. 2017; Basson et al. 2023). The presence of 4‐hydroxybenzoic acid in the extract highlighted its antiproliferative and proapoptotic activities (Seidel et al. 2016; Myint et al. 2021). Borneol enhanced the effectiveness of chemotherapy in resistant lung cancer cases and improved the cellular uptake of therapeutic agents, indicating its potential in overcoming breast cancer resistance to tamoxifen (Li et al. 2023). Moreover, borneol has been identified as a sensitizer of glioma cells to radiation, achieved through the induction of autophagy mediated by inhibition of the mTORC1/eIF4E/HIF‐1α regulatory axis (Li et al. 2021; Lin et al. 2024). β‐Eudesmol inhibited breast cancer cell proliferation, induced ferroptosis, and showed cytotoxic effects on cholangiocarcinoma, offering a novel treatment strategy (Li et al. 2024). Camphor, known for its analgesic properties, also demonstrated potential as an anticancer agent (Singh et al. 2023). The extract's rich phytochemical composition and diverse mechanisms inherent to *A. herba‐alba* support the observed anticancer effects elucidated in our study.

The differential cytotoxicity against a panel of CRC cell lines, as evidenced by varying IC_50_ values, underscores the extract's selective efficacy, which could be attributed to these cell lines' distinct genetic and molecular profiles.

Recent scientific studies have shown that *A. herba‐alba* has a strong cytotoxic effect on several human cancer cell lines, encompassing kidney carcinoma, lung cancer, and most importantly, colon cancer (Khlifi et al. 2013; Tilaoui et al. 2015; Ali, Saeed, and Omear 2021; Amkiss et al. 2022; Jakovljević et al. 2023). While several studies investigated the effects of Artemisia species on human colon cancer cells (Khlifi et al. 2013; Ali, Saeed, and Omear 2021; Jakovljević et al. 2023), our study uniquely focused on assessing the cytotoxic properties across a diverse range of colorectal cancer cells including the isogenic cell lines HCT116 and RKO, which are differentiated only by their p53 status.

The various responses observed among CRC cell lines when treated with *A. herba‐alba* , with a particular emphasis on the resistance observed in SW480 and SW620 cells, provide valuable insights into the intricate nature of cancer cell biology and the significance of specific genetic and phenotypic markers in terms of therapeutic resistance.

It is suggested that the resistance displayed by these cell lines is associated with their increased levels of CXCR4, a crucial element in the CXCR4/CXCL12 axis known to support resistance mechanisms to treatment (Chatterjee, Behnam Azad, and Nimmagadda 2014; Kim, Min, and Park 2020; Hjazi et al. 2023).

Moreover, the observed variation in IC_50_ values across various CRC cell lines when exposed to the methanolic extract of *A. herba‐alba* underscores the genetic and phenotypic diversity in cancer cells. This finding implies that cancer cells exhibit distinct biological responses, which could provide valuable insights for developing treatment approaches. Both p53 positive and negative variants of HCT116 and RKO cell lines are found sensitive to *A. herba‐alba*, even though the p53 positive variant of HCT116 has a slightly higher IC_50_ value. Such evidence indicates that the extract might exert its effects via a pathway other than the p53 pathway.

Furthermore, we investigated the effect of the extract on the cell cycle dynamics of colorectal cancer cells. Chemotherapy has conventionally sought to cause the distribution of the cell cycle to interfere with the synthesis of RNA or DNA, thereby impeding the division of malignant cells at different phases of the cell cycle and ultimately resulting in the reduction of tumor size. *A. herba‐alba* was found to cause a significant arrest in the cell cycle in all the CRC cell lines studied, primarily at the G2‐M phase. The G2‐M phase checkpoint is essential for preventing cells from entering mitosis with malfunctioning DNA, thereby providing an opportunity to repair or remove the damage (Stark and Taylor 2004; Kalsbeek and Golsteyn 2017).

Generally, cancer cells with mutant or absent p53 tend to be more resistant to traditional chemotherapies, considering the crucial significance of the p53 protein in preserving genomic integrity and its involvement in regulating G1/S and G2/M phase checkpoints (Mehta, Goldfarb, and Zinterhofer 2002; Maurer et al. 2011). Surprisingly, the cell lines (HCT116 and RKO) with p53‐negative genotypes showed similar or relatively higher response (Table 2), which is consistent with the results of previous study suggesting that cell cycle arrest and death may occur without p53, specifically through DNA damage‐dependent G2 checkpoints (Jaiswal, Oh, and DePamphilis 2020). These findings suggest that *A. herba‐alba* may directly impact crucial regulators of the G2‐M phase transition, namely Cyclin B1 and CDK1. Consequently, this leads to a G2‐M phase arrest in all CRC cell lines that were examined, regardless of their p53 status. Indeed, western blot analysis demonstrated that *A. herba‐alba* extract effectively decreased Cyclin B1 and CDK1 levels.

The cellular response to *A. herba‐alba* extract was investigated using the annexin V‐FITC/PI assay, which provided additional insights into the underlying mechanism of cell death induced by the treatment. This experiment demonstrated the activation of both apoptotic and necrotic pathways in response to *A. herba‐alba* . Caco‐2, RKO^+/+^p53, and RKO^−/−^p53 cells exhibited a preference for apoptosis, but DLD1, HCT116^+/+^p53, and HCT116−/−p53 cells exhibited an equal involvement of both apoptosis and necrosis.

The evaluation of the pharmacological potential of *A. herba‐alba* against CRC includes a critical examination of its impact on the PI3K/AKT/mTOR signaling pathway. This route is crucial in controlling various cellular processes, such as cell division, survival, and metabolism. It is often disrupted in CRC, which leads to cancer progression and resistance to treatment. The results of our study demonstrate a complex regulation of this network by *A. herba‐alba* , as indicated by the varying effects on the phosphorylation levels of AKT and mTOR in distinct CRC cell lines. Specifically, a notable decrease in the phosphorylation of AKT and mTOR in cell lines such as RKO^+/+^p53, RKO^−/−^p53, and Caco2 was observed. This finding suggests a strong suppression of these pathways' signaling cascades. In contrast, it was primarily observed that the impact on AKT phosphorylation was reduced in HCT116^−/−^p53 and DLD1 cell lines, whereas mTOR phosphorylation remained essentially unchanged. The differential regulation seen in this study indicates that *A. herba‐alba* exhibits a selective impact on the PI3K/AKT/mTOR pathway, which may differ among cell lines due to their unique genetic and phenotypic characteristics. Additionally, it is noteworthy that various other species within the genus Artemisia have demonstrated the capacity to suppress the PI3K/AKT/mTOR pathway (Kim et al. 2020; Su et al. 2022). The capacity of *A. herba‐alba* to selectively regulate this crucial signaling pathway provides significant knowledge regarding its mode of operation and highlights its potential as a focused therapeutic intervention in treating colorectal cancer. Further investigation is necessary to elucidate the precise mechanism of action and identify the extract's active constituents.

## 5 Conclusions

Our study on *Artemisia herba‐alba* emphasizes its potential as an anticancer agent for CRC. Our findings demonstrate its cytotoxic effects, ability to induce apoptosis, ability to arrest the cell cycle, and modulation of the PI3K/AKT/mTOR pathway in different CRC cell lines. Significantly, its influence goes beyond relying on p53, suggesting a wider therapeutic significance. Further investigation is warranted to elucidate the molecular mechanisms and clinical efficacy of *A. herba‐alba* in the context of cancer treatment.

## Author Contributions

**Lara J. Bou Malhab:** methodology (lead), writing – original draft (equal). **Amani A. Harb:** methodology (equal). **Leen Eldohaji:** methodology (equal), validation (lead). **Jalal Taneera:** writing – review and editing (equal). **Hamza M. Al‐Hroub:** data curation (equal), methodology (equal). **Ahmad Abuhelwa:** visualization (equal), writing – review and editing (equal). **Karem H. Alzoubi:** formal analysis (equal), writing – review and editing (equal). **Bashaer Abu‐Irmaileh:** methodology (equal). **Mohammad Hudaib:** data curation (equal), formal analysis (equal). **Jehad Almaliti:** formal analysis (equal). **Wael M. Abdel‐Rahman:** resources (equal), writing – review and editing (equal). **Abdallah Shanableh:** resources (equal). **Mohammad H. Semreen:** data curation (equal), writing – review and editing (equal). **Waseem El‐Huneidi:** data curation (equal), writing – review and editing (equal). **Eman Abu‐Gharbieh:** conceptualization (equal), writing – review and editing (equal). **Yasser Bustanji:** conceptualization (equal), supervision (equal).

## Consent

The authors have nothing to report.

## Conflicts of Interest

The authors declare no conflicts of interest.

## Supporting information


**Figure S1.** Gas chromatography–mass spectrometry analysis in the methanolic extract of *A. herba‐alba* (after chemical derivatization).

**Table S1.** Phytochemical profiling of *A. herba‐alba* methanolic extract.

## Data Availability

All data generated or analyzed during this study are included in this article or in the Supporting Information appended to it. Further inquiries can be directed to the corresponding author.

## References

[fsn34715-bib-0001] Abu‐Gharbieh, E. , N. G. Shehab , I. M. Almasri , and Y. Bustanji . 2019. “Antihyperuricemic and Xanthine Oxidase Inhibitory Activities of Tribulus Arabicus and Its Isolated Compound, Ursolic Acid: In Vitro and In Vivo Investigation and Docking Simulations.” PLoS One 13, no. 8: e0202572. 10.1371/journal.pone.0202572. PMC6095567 30114281

[fsn34715-bib-0002] Abu‐Rish, E. Y. , V. Kasabri , M. M. Hudaib , et al. 2016. “Evaluation of Antiproliferative Activity of Some Traditional Anticancer Herbal Remedies From Jordan.” Tropical Journal of Pharmaceutical Research 15, no. 3: 469–474. 10.4314/tjpr.v15i3.6.

[fsn34715-bib-0003] Ahmed, A. H. , M. Ejo , T. Feyera , D. Regassa , B. Mummed , and S. A. Huluka . 2020. “In Vitro Anthelmintic Activity of Crude Extracts of *Artemisia herba‐alba* and *Punica granatum* Against Haemonchus Contortus.” Journal of Parasitology Research 2020: 1–7. 10.1155/2020/4950196. PMC7204145 32411422

[fsn34715-bib-0004] Al‐Eisawi, Z. , S. M. Abderrahman , Y. M. S. Abdelrahim , R. Al‐Abbassi , and Y. K. Bustanji . 2022. “Anastatica Hierochuntica Extracts: Promising, Safe and Selective Anti‐Cancer Agents.” Natural Products Journal 12, no. 1: 78–87. 10.2174/2210315510999200914153725.

[fsn34715-bib-0005] Alhourani, N. T. , M. M. D. Hudaib , Y. K. Bustanji , R. Alabbassi , and V. Kasabri . 2020. “Chemical Composition of Essential Oil and Screening of Antiproliferative Activity of Paronychia Argentea Lam. Aerial Parts: An Ethno‐Medicinal Plant From Jordan.” Jordan Journal of Pharmaceutical Sciences 13, no. 3: 291–301.

[fsn34715-bib-0006] Ali, A. N. M. , N. A. H. A. A. H. Saeed , and H. A. Omear . 2021. “The Anticancer Properties of *Artemisia aucheri* Boiss Extract on HT29 Colon Cancer Cells.” Journal of Gastrointestinal Cancer 52, no. 1: 113–119. 10.1007/s12029-019-00354-2. 31907764

[fsn34715-bib-0007] Al‐Qbilat, S. , and O. Atrooz . 2024. “In Vitro Validation of Biological and Cytotoxic Activity of Methanol Extract of Jordanian *Artemisia herba‐aaba* .” Journal of Microbiology, Biotechnology and Food Sciences 13, no. 4: e10247. 10.55251/jmbfs.10247.

[fsn34715-bib-0008] Altay, A. , E. Yeniceri , P. Taslimi , T. Taskin‐Tok , M. A. Yilmaz , and E. Koksal . 2022. “LC‐MS/MS Analysis and Diverse Biological Activities of *Hypericum scabrum* L.: In Vitro and In Silico Research.” South African Journal of Botany 150: 940–955. 10.1016/j.sajb.2022.08.032.

[fsn34715-bib-0009] Althaher, A. R. , S. A. Oran , and Y. K. Bustanji . 2020. “Phytochemical Analysis, in Vitro Assessment of Antioxidant Properties and Cytotoxic Potential of *Ruta chalepensis* L. Essential Oil.” Journal of Essential Oil‐Bearing Plants 23, no. 6: 1409–1421. 10.1080/0972060X.2020.1871078.

[fsn34715-bib-0010] Althaher, A. R. , S. A. Oran , and Y. K. Bustanji . 2021. “Chemical Composition, In Vitro Evaluation of Antioxidant Properties and Cytotoxic Activity of the Essential Oil From Calamintha incana (Sm.) Helder (Lamiaceae).” Tropical Journal of Natural Product Research 5, no. 8: 1333–1339. 10.26538/tjnpr/v5i8.2.

[fsn34715-bib-0011] Althaher, A. R. , S. A. Oran , and Y. K. Bustanji . 2022. “Induction of Apoptosis by *Ruta chalepensis* L. Essential Oil in Human Breast Cancer Cells (MCF‐7).” Journal of Pharmacy and Pharmacognosy Research 10, no. 1: 73–83.

[fsn34715-bib-0012] Amkiss, S. , M. Bakha , O. Belmehdi , F. Carmona‐Espinazo , J. B. López‐Sáez , and M. Idaomar . 2022. “Antioxidant Activity and Polyphenols Content of *Artemisia herba‐alba* Extract and Their Cytotoxicity Against Human Lung Cancer Cells NCI‐N417.” Journal of Herbs Spices & Medicinal Plants 28, no. 4: 337–350. 10.1080/10496475.2022.2064954.

[fsn34715-bib-0013] Bajes, H. , S. Oran , and Y. Bustanji . 2023. “Phytochemical Analysis, in Vitro Assessment of Antioxidant Properties and Cytotoxic Potential of *Thymus capitatus* Essential Oil.” Research Journal of Pharmacy and Technology 16, no. 3: 1100–1108. 10.52711/0974-360X.2023.00183.

[fsn34715-bib-0014] Bajes, H. R. , I. Almasri , and Y. Bustanji . 2020. “Plant Products and Their Inhibitory Activity Against Pancreatic Lipase.” Revista Brasileira de Farmacognosia 30, no. 3: 321–330. 10.1007/s43450-020-00055-z.

[fsn34715-bib-0015] Bajes, H. R. , S. A. Oran , and Y. K. Bustanji . 2021. “Chemical Composition and Antiproliferative and Antioxidant Activities of Methanolic Extract of Alcea Setosa A. Malvaceae.” Research Journal of Pharmacy and Technology 14, no. 12: 6447–6454. 10.52711/0974-360X.2021.01115.

[fsn34715-bib-0016] Bajes, H. R. , S. A. Oran , and Y. K. Bustanji . 2022. “Chemical Composition and Antiproliferative and Antioxidant Activities of Essential Oil From *Juniperus phoenicea* L. Cupressaceae.” Research Journal of Pharmacy and Technology 15, no. 1: 153–159. 10.52711/0974-360X.2022.00025.

[fsn34715-bib-0017] Basson, C. , J. C. Serem , Y. N. Hlophe , and P. Bipath . 2023. “An In Vitro Investigation of l‐Kynurenine, Quinolinic Acid, and Kynurenic Acid on B16 F10 Melanoma Cell Cytotoxicity and Morphology.” Cell Biochemistry and Function 41, no. 7: 912–922. 10.1002/cbf.3843. 37661337

[fsn34715-bib-0018] Bayrakçeken Güven, Z. , I. Saracoglu , A. Nagatsu , M. A. Yilmaz , and A. A. Basaran . 2023. “Anti‐Tyrosinase and Antimelanogenic Effect of Cinnamic Acid Derivatives From *Prunus mahaleb* L.: Phenolic Composition, Isolation, Identification and Inhibitory Activity.” Journal of Ethnopharmacology 310: 116378. 10.1016/j.jep.2023.116378. 36924865

[fsn34715-bib-0019] Benchohra, M. , A. Ahmed , and O. Merah . 2023. “Relationship Between Variations in Ecological Conditions and the Dynamics of Intra‐Specific Morphological Diversity of *Artemisia herba‐alba* Asso in Algeria.” Ekologia Bratislava 42, no. 3: 209–217. 10.2478/eko-2023-0024.

[fsn34715-bib-0020] Biswas, S. , N. D. Reddy , B. S. Jayashree , and C. M. Rao . 2018. “Evaluation of Novel 3‐Hydroxyflavone Analogues as HDAC Inhibitors Against Colorectal Cancer.” Advances in Pharmacological Sciences 2018: 1–14. 10.1155/2018/4751806. PMC6327263 30687400

[fsn34715-bib-0021] Boğa, M. , H. Alkan , A. Ertaş , et al. 2016. “Phytochemical Profile and Some Biological Activities of Three Centaurea Species From Turkey.” Tropical Journal of Pharmaceutical Research 15, no. 9: 1865–1875. 10.4314/tjpr.v15i9.8.

[fsn34715-bib-0022] Bou Malhab, L. J. , K. Bajbouj , N. G. Shehab , et al. 2023. “Potential Anticancer Properties of *Calotropis procera* : An Investigation on Breast and Colon Cancer Cells.” Heliyon 9, no. 6: e16706. 10.1016/j.heliyon.2023.e16706. 37332907 PMC10272338

[fsn34715-bib-0023] Bustanji, Y. , M. Hudaib , K. Tawaha , et al. 2011. “In Vitro Xanthine Oxidase Inhibition by Selected Jordanian Medicinal Plants.” Jordan Journal of Pharmaceutical Sciences 4, no. 1: 49–55.

[fsn34715-bib-0024] Bustanji, Y. , N. Quqazeh , M. Mohammad , et al. 2023. “Screening of Some Medicinal Plant Extracts for Their Lipoprotein Lipase Inhibition Activity.” Research Journal of Pharmacy and Technology 16, no. 10: 4786–4790. 10.52711/0974-360X.2023.00776.

[fsn34715-bib-0025] Ceylan, R. , G. Zengin , M. F. Mahomoodally , et al. 2021. “Enzyme Inhibition and Antioxidant Functionality of Eleven Inula Species Based on Chemical Components and Chemometric Insights.” Biochemical Systematics and Ecology 95: 104225. 10.1016/j.bse.2021.104225.

[fsn34715-bib-0026] Chatterjee, S. , B. Behnam Azad , and S. Nimmagadda . 2014. “The Intricate Role of CXCR4 in Cancer.” Advances in Cancer Research, 124, 31–82. 10.1016/B978-0-12-411638-2.00002-1. 25287686 PMC4322894

[fsn34715-bib-0027] Chen, D. , F. Ma , X. H. Liu , R. Cao , and X. Z. Wu . 2016. “Anti‐Tumor Effects of Ephedrine and Anisodamine on SKBR3 Human Breast Cancer Cell Line.” African Journal of Traditional, Complementary, and Alternative Medicines 13, no. 1: 25–32. 10.4314/ajtcam.v13i1.4.

[fsn34715-bib-0028] Dawra, M. , J. Bouajila , M. El Beyrouthy , P. Taillandier , N. Nehme , and Y. El Rayess . 2024. “Phytochemical Profile, GC‐MS Profiling and In Vitro Evaluation of Some Biological Applications of the Extracts of *Origanum syriacum* L. and Cousinia Libanotica D.C.” Plants 13, no. 1: 137. 38202445 10.3390/plants13010137 PMC10780604

[fsn34715-bib-0029] Dmour, S. M. , S. A. M. Saghir , S. Abushattal , et al. 2024. “Biological Activities and Chemical Composition of Essential Oil Isolated From *Artemisia herba‐alba* .” Electronic Journal of General Medicine 21, no. 1: em569. 10.29333/ejgm/14161.

[fsn34715-bib-0030] El Hajli, F. , M. R. Kachmar , A. Assouguem , et al. 2024. “Phytochemical Analysis, In Vitro Antioxidant and Antifungal Activities of Extracts and Essential Oil Derived From *Artemisia herba‐alba* Asso.” Open Chemistry 22, no. 1: 20230200. 10.1515/chem-2023-0200.

[fsn34715-bib-0031] Elbahaie, E. S. , R. L. El Gamal , G. M. Fathy , et al. 2023. “The Controverted Therapeutic Efficacy of Allium Sativum and *Artemisia herba‐alba* Extracts on Cryptosporidium‐Infected Mice.” Journal of Infection in Developing Countries 17, no. 6: 732–743. 10.3855/jidc.17360. 37406057

[fsn34715-bib-0032] Elwardani, H. , A. Oubihi , S. Haida , R. Ez‐Zriouli , K. E. Kabous , and M. Ouhssine . 2024. “Seasonal Variation in Essential Oil Composition of *Artemisia herba‐alba* and Their Effects on Antioxidant, Antibacterial, and Antifungal Activities.” Chemical Data Collections 50: 101118. 10.1016/j.cdc.2024.101118.

[fsn34715-bib-0033] Fang, X. , J. Song , K. Zhou , et al. 2023. “Molecular Mechanism Pathways of Natural Compounds for the Treatment of Non‐Alcoholic Fatty Liver Disease.” Molecules 28, no. 15: 5645. 10.3390/molecules28155645. 37570615 PMC10419790

[fsn34715-bib-0034] Gmeiner, W. H. 2024. “Recent Advances in Therapeutic Strategies to Improve Colorectal Cancer Treatment.” Cancers 16, no. 5: 1029. 10.3390/cancers16051029. 38473386 PMC10930828

[fsn34715-bib-0035] Harb, A. A. , Y. K. Bustanji , and S. S. Abdalla . 2018. “Hypocholesterolemic Effect of β‐Caryophyllene in Rats Fed Cholesterol and Fat Enriched Diet.” Journal of Clinical Biochemistry and Nutrition 62, no. 3: 230–237. 29892161 10.3164/jcbn.17-3 PMC5990408

[fsn34715-bib-0036] Hasan, A. , P. Biswas , T. A. Bondhon , et al. 2022. “Can *Artemisia herba‐alba* be Useful for Managing COVID‐19 and Comorbidities?” Molecules 27, no. 2: 492. 10.3390/molecules27020492. 35056809 PMC8779608

[fsn34715-bib-0037] Hernandez‐Martinez, J. M. , C. M. Forrest , L. G. Darlington , R. A. Smith , and T. W. Stone . 2017. “Quinolinic Acid Induces Neuritogenesis in SH‐SY5Y Neuroblastoma Cells Independently of NMDA Receptor Activation.” European Journal of Neuroscience 45, no. 5: 700–711. 10.1111/ejn.13499. 27973747

[fsn34715-bib-0038] Hjazi, A. , F. Nasir , R. Noor , et al. 2023. “The Pathological Role of C‐X‐C Chemokine Receptor Type 4 (CXCR4) in Colorectal Cancer (CRC) Progression; Special Focus on Molecular Mechanisms and Possible Therapeutics.” Pathology Research and Practice 248: 154616. 10.1016/j.prp.2023.154616. 37379710

[fsn34715-bib-0039] Jaiswal, S. K. , J. J. Oh , and M. L. DePamphilis . 2020. “Cell Cycle Arrest and Apoptosis Are Not Dependent on p53 Prior to p53‐Dependent Embryonic Stem Cell Differentiation.” Stem Cells 38, no. 9: 1091–1106. 10.1002/stem.3199. 32478947

[fsn34715-bib-0040] Jakovljević, M. R. , M. Milutinović , P. Djurdjević , Ž. Todorović , M. Stanković , and O. Milošević‐Djordjević . 2023. “Cytotoxic and Apoptotic Activity of Acetone and Aqueous *Artemisia vulgaris* L. and Artemisia Alba Turra Extracts on Colorectal Cancer Cells.” European Journal of Integrative Medicine 57: 102204. 10.1016/j.eujim.2022.102204.

[fsn34715-bib-0041] Kalsbeek, D. , and R. M. Golsteyn . 2017. “G2/M‐Phase Checkpoint Adaptation and Micronuclei Formation as Mechanisms That Contribute to Genomic Instability in Human Cells.” International Journal of Molecular Sciences 18, no. 11: 2344. 10.3390/ijms18112344. 29113112 PMC5713313

[fsn34715-bib-0042] Kasabri, V. , F. U. Afifi , R. Abu‐Dahab , et al. 2014. “In Vitro Modulation of Metabolic Syndrome Enzymes and Proliferation of Obesity Related‐Colorectal Cancer Cell Line Panel by Salvia Species From Jordan.” Revue Roumaine de Chimie 59, no. 8: 693–705.

[fsn34715-bib-0043] Kasabri, V. , E. K. Al‐Hallaq , Y. K. Bustanji , K. K. Abdul‐Razzak , I. F. Abaza , and F. U. Afifi . 2017. “Antiobesity and Antihyperglycaemic Effects of *Adiantum capillus‐veneris* Extracts: In Vitro and In Vivo Evaluations.” Pharmaceutical Biology 55, no. 1: 164–172. 10.1080/13880209.2016.1233567. 27663206 PMC7011982

[fsn34715-bib-0044] Khlifi, D. , R. M. Sghaier , S. Amouri , D. Laouini , M. Hamdi , and J. Bouajila . 2013. “Composition and Anti‐Oxidant, Anti‐Cancer and Anti‐Inflammatory Activities of *Artemisia herba‐alba* , *Ruta chalpensis* L. and *Peganum harmala* L.” Food and Chemical Toxicology 55: 202–208. 10.1016/j.fct.2013.01.004. 23333573

[fsn34715-bib-0045] Kim, B. , Y. H. Min , and B. Park . 2020. “Minecoside Modulates Cell Invasion via Regulation of CXCR4 Expression in Breast and Colon Cancer Cells.” Planta Medica 86, no. 5: 331–337. 10.1055/a-1107-3272. 32016931

[fsn34715-bib-0046] Kim, J. , K. H. Jung , J. G. Choi , M. S. Oh , and S. S. Hong . 2020. “Artemisiae Iwayomogii Herba Inhibits Growth, Motility, and the PI3K/AKT/mTOR Signaling Pathway in Hepatocellular Carcinoma Cells.” Planta Medica 86, no. 10: 717–727. 10.1055/a-1167-4284. 32428938

[fsn34715-bib-0047] Li, J. X. , J. J. Wang , R. Ma , et al. 2023. “Sensitizing Effect of d‐Borneol on Cisplatin‐Resistant NSCLC Based on Transcriptomics and Its Mechanism.” Chinese Pharmacological Bulletin 39, no. 6: 1105–1114. 10.12360/CPB202205013.

[fsn34715-bib-0048] Li, Q. , L. Xia , C. Sun , et al. 2021. “Role of Borneol Induced Autophagy in Enhancing Radiosensitivity of Malignant Glioma.” Frontiers in Oncology 11: 749987. 10.3389/fonc.2021.749987. 34917504 PMC8668811

[fsn34715-bib-0049] Li, Z. , J. Li , X. Liu , Y. Liu , H. Chen , and X. Sun . 2024. “β‐Eudesmol Inhibits Cell Proliferation and Induces Ferroptosis via Regulating MAPK Signaling Pathway in Breast Cancer.” Toxicon 237: 107529. 10.1016/j.toxicon.2023.107529. 38030095

[fsn34715-bib-0050] Lin, L. , J. Luo , Z. Wang , and X. Cai . 2024. “Borneol Promotes Autophagic Degradation of HIF‐1α and Enhances Chemotherapy Sensitivity in Malignant Glioma.” PeerJ 12: e16691. 10.7717/peerj.16691. 38188151 PMC10771087

[fsn34715-bib-0051] Marabotto, E. , S. Kayali , S. Buccilli , et al. 2022. “Colorectal Cancer in Inflammatory Bowel Diseases: Epidemiology and Prevention: A Review.” Cancers 14, no. 17: 4254. 10.3390/cancers14174254. 36077786 PMC9454776

[fsn34715-bib-0052] Maurer, M. , O. Komina , A. Składanowski , and J. Wȩsierska‐Ga̧dek . 2011. “Triazoloacridone C‐1305 Abrogates the Restriction Checkpoint in Cells Lacking Functional p53 and Promotes Their Accumulation in the G2/M Phase of the Cell Cycle.” Journal of Experimental Therapeutics and Oncology 9, no. 1: 5–15. 21275261

[fsn34715-bib-0053] Mehta, K. U. , M. A. Goldfarb , and L. J. Zinterhofer . 2002. “Absent p53 Protein in Colorectal Tumor Cells Reflects Poor Survival.” Journal of Applied Research 2, no. 3: XV–XVI.

[fsn34715-bib-0054] Memarzia, A. , S. Saadat , F. Asgharzadeh , S. Behrouz , G. Folkerts , and M. H. Boskabady . 2023. “Therapeutic Effects of Medicinal Plants and Their Constituents on Lung Cancer, In Vitro, In Vivo and Clinical Evidence.” Journal of Cellular and Molecular Medicine 27, no. 19: 2841–2863. 10.1111/jcmm.17936. 37697969 PMC10538270

[fsn34715-bib-0055] Mia, M. A. R. , D. Dey , M. R. Sakib , et al. 2023. “The Efficacy of Natural Bioactive Compounds Against Prostate Cancer: Molecular Targets and Synergistic Activities.” Phytotherapy Research 37, no. 12: 5724–5754. 10.1002/ptr.8017. 37786304

[fsn34715-bib-0056] Mohamed, A. E. H. H. , M. A. El‐Sayed , M. E. Hegazy , S. E. Helaly , A. M. Esmail , and N. S. Mohamed . 2010. “Chemical Constituents and Biological Activities of *Artemisia herba‐alba* .” Records of Natural Products 4, no. 1: 1–25.

[fsn34715-bib-0057] Mohammad, M. K. , I. M. Almasri , K. Tawaha , et al. 2010. “Antioxidant, Antihyperuricemic and Xanthine Oxidase Inhibitory Activities of Hyoscyamus Reticulatus.” Pharmaceutical Biology 48, no. 12: 1376–1383. 10.3109/13880209.2010.483521. 20738177

[fsn34715-bib-0058] Mrabti, H. N. , N. E. Hachlafi , S. H. Al‐Mijalli , et al. 2023. “Phytochemical Profile, Assessment of Antimicrobial and Antioxidant Properties of Essential Oils of *Artemisia herba‐alba* Asso., and *Artemisia dracunculus* L.: Experimental and Computational Approaches.” Journal of Molecular Structure 1294: 136479. 10.1016/j.molstruc.2023.136479.

[fsn34715-bib-0059] Murphy, C. C. , and T. A. Zaki . 2024. “Changing Epidemiology of Colorectal Cancer—Birth Cohort Effects and Emerging Risk Factors.” Nature Reviews Gastroenterology and Hepatology 21, no. 1: 25–34. 10.1038/s41575-023-00841-9. 37723270

[fsn34715-bib-0060] Myint, O. , S. Wattanapongpitak , B. Supawat , et al. 2021. “Protein Binding of 4‐Hydroxybenzoic Acid and 4‐Hydroxy‐3‐Methoxybenzoic Acid to Human Serum Albumin and Their Anti‐Proliferation on Doxorubicin‐Sensitive and Doxorubicin‐Resistant Leukemia Cells.” Toxicology Reports 8: 1381–1388. 10.1016/j.toxrep.2021.07.001. 34285884 PMC8278208

[fsn34715-bib-0061] Ogunlakin, A. D. , M. A. Sonibare , O. E. Yeye , et al. 2023. “Isolation and Characterization of Novel Hydroxyflavone From *Kigelia africana* (Lam.) Benth. Fruit Ethyl Acetate Fraction Against CHO 1 and HeLa Cancer Cell Lines: In Vitro and In Silico Studies.” Journal of Molecular Structure 1282: 135180. 10.1016/j.molstruc.2023.135180.

[fsn34715-bib-0062] Ouahdani, K. E. , I. Es‐Safi , H. Mechchate , et al. 2021. “Thymus Algeriensis and *Artemisia herba‐alba* Essential Oils: Chemical Analysis, Antioxidant Potential and In Vivo Anti‐Inflammatory, Analgesic Activities, and Acute Toxicity.” Molecules 26, no. 22: 6780. 10.3390/molecules26226780. 34833872 PMC8625911

[fsn34715-bib-0063] Paul, T. , K. Pathak , R. Saikia , U. Gogoi , J. J. Sahariah , and A. Das . 2024. “The Role of Medicinal Plants in the Treatment and Management of Type 2 Diabetes.” Current Traditional Medicine 10, no. 2: 83–96. 10.2174/2215083809666230223164613.

[fsn34715-bib-0064] Rakotondrabe, T. F. , M. X. Fan , F. W. Muema , and M. Q. Guo . 2023. “Modulating Inflammation‐Mediated Diseases via Natural Phenolic Compounds Loaded in Nanocarrier Systems.” Pharmaceutics 15, no. 2: 699. 10.3390/pharmaceutics15020699. 36840021 PMC9964760

[fsn34715-bib-0065] Sayed, U. , M. Hudaib , A. Issa , K. Tawaha , and Y. Bustanji . 2021. “Plant Products and Their Inhibitory Activity Against Xanthine Oxidase.” Farmácia 69, no. 6: 1042–1052. 10.31925/farmacia.2021.6.4.

[fsn34715-bib-0066] Seidel, C. , M. Schnekenburger , A. Mazumder , et al. 2016. “4‐Hydroxybenzoic Acid Derivatives as HDAC6‐Specific Inhibitors Modulating Microtubular Structure and HSP90α Chaperone Activity Against Prostate Cancer.” Biochemical Pharmacology 99: 31–52. 10.1016/j.bcp.2015.11.005. 26549368

[fsn34715-bib-0067] Selek, S. , I. Koyuncu , H. G. Caglar , et al. 2018. “The Evaluation of Antioxidant and Anticancer Effects of *Lepidium sativum* subsp Spinescens L. Methanol Extract on Cancer Cells.” Cellular and Molecular Biology 6, no. 3: 72–80. 10.14715/cmb/2018.64.3.12. 29506633

[fsn34715-bib-0068] Singh, H. , R. Kumar , A. Mazumder , R. K. Yadav , B. Chau‐Han , and M. M. Abdulah . 2023. “Camphor and Menthol as Anticancer Agents: Synthesis, Structure‐Activity Relationship and Interaction With Cancer Cell Lines.” Anti‐Cancer Agents in Medicinal Chemistry 23, no. 6: 614–623. 10.2174/1871520622666220810153735. 35950244

[fsn34715-bib-0069] Sirajudeen, F. , L. J. Bou Malhab , Y. Bustanji , et al. 2024. “Exploring the Potential of Rosemary Derived Compounds (Rosmarinic and Carnosic Acids) as Cancer Therapeutics: Current Knowledge and Future Perspectives.” Biomolecules & Therapeutics 32, no. 1: 38–55. 10.4062/biomolther.2023.054. 38148552 PMC10762267

[fsn34715-bib-0070] Souhila, T. , B. Fatma Zohra , and H. S. Tahar . 2019. “Identification and Quantification of Phenolic Compounds of *Artemisia herba‐alba* at Three Harvest Time by HPLC–ESI–Q‐TOF–MS.” International Journal of Food Properties 22, no. 1: 843–852. 10.1080/10942912.2019.1614051.

[fsn34715-bib-0071] Stark, G. R. , and W. R. Taylor . 2004. “Analyzing the G2/M Checkpoint.” Methods in Molecular Biology 280: 51–82. 15187249 10.1385/1-59259-788-2:051

[fsn34715-bib-0072] Su, S. H. , N. Sundhar , W. W. Kuo , et al. 2022. “Artemisia Argyi Extract Induces Apoptosis in Human Gemcitabine‐Resistant Lung Cancer Cells via the PI3K/MAPK Signaling Pathway.” Journal of Ethnopharmacology 299: 115658. 10.1016/j.jep.2022.115658. 36075273

[fsn34715-bib-0073] Tarawneh, N. , L. Hamadneh , B. Abu‐Irmaileh , Z. Shraideh , Y. Bustanji , and S. Abdalla . 2023. “Berberine Inhibited Growth and Migration of Human Colon Cancer Cell Lines by Increasing Phosphatase and Tensin and Inhibiting Aquaporins 1, 3 and 5 Expressions.” Molecules 28, no. 9: 3823. 10.3390/molecules28093823. 37175233 PMC10180100

[fsn34715-bib-0074] Tilaoui, M. , H. A. Mouse , A. Jaafari , and A. Zyad . 2015. “Comparative Phytochemical Analysis of Essential Oils From Different Biological Parts of Artemisia Herba Alba and Their Cytotoxic Effect on Cancer Cells.” PLoS One 10, no. 7: e0131799. 10.1371/journal.pone.0131799. 26196123 PMC4510584

[fsn34715-bib-0075] Ullah, R. , G. Ali , A. Rasheed , et al. 2022. “The 7‐Hydroxyflavone Attenuates Chemotherapy‐Induced Neuropathic Pain by Targeting Inflammatory Pathway.” International Immunopharmacology 107: 108674. 10.1016/j.intimp.2022.108674. 35276461

[fsn34715-bib-0076] Wang, M. , X. Liu , T. Chen , et al. 2022. “Inhibition and Potential Treatment of Colorectal Cancer by Natural Compounds via Various Signaling Pathways.” Frontiers in Oncology 12: 956793. 10.3389/fonc.2022.956793. 36158694 PMC9496650

[fsn34715-bib-0077] Witayateeraporn, W. , B. Hardianti , and V. Pongrakhananon . 2024. “Comprehensive Review of Bcl‐2 Family Proteins in Cancer Apoptosis: Therapeutic Strategies and Promising Updates of Natural Bioactive Compounds and Small Molecules.” Phytotherapy Research 38: 2249–2275. 10.1002/ptr.8157. 38415799

[fsn34715-bib-0078] Yener, I. , M. A. Yilmaz , O. T. Olmez , et al. 2020. “A Detailed Biological and Chemical Investigation of Sixteen Achillea Species' Essential Oils via Chemometric Approach.” Chemistry and Biodiversity 17, no. 3: e1900484. 10.1002/cbdv.201900484. 31999042

[fsn34715-bib-0079] Yousef, I. , S. Oran , M. Alqaraleh , and Y. Bustanji . 2021. “Evaluation of Cytotoxic, Antioxidant and Antibacterial Activities of Origanum Dayi, Salvia Palaestina and Bongardia Chrysogonum Plants Growing Wild in Jordan.” Tropical Journal of Natural Product Research 5, no. 1: 66–70. 10.26538/tjnpr/v5i1.7.

