# Supplementary material for: Exploring the Anticancer Effect of Artemisia herba‐alba on Colorectal Cancer: Insights From Eight Colorectal Cancer Cell Lines
Source: Food Sci Nutr. 2024 Dec 31;13(1):e4715. doi: 10.1002/fsn3.4715 (PMC11717013; doi:10.1002/fsn3.4715)
Supplement: Supplementary file 1 — Figure S1. Gas chromatography–mass spectrometry analysis in the methanolic extract of A. herba‐alba (after chemical derivatization). [file FSN3-13-e4715-s001.docx]

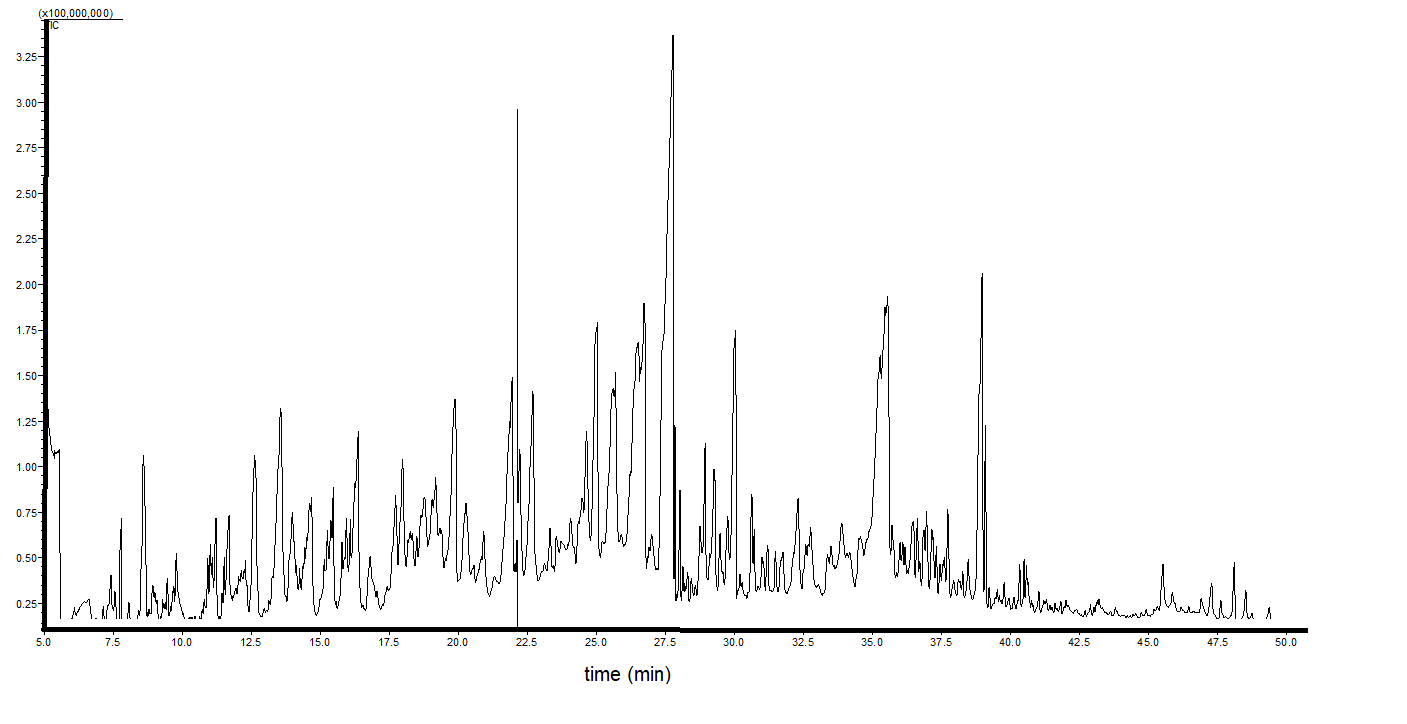


**Figure S1:** Gas chromatography-mass spectrometry analysis in the methanolic extract of *A. herba-alba* (after chemical derivatization).
