# Supplementary material for: Exploring the Anticancer Effect of Artemisia herba‐alba on Colorectal Cancer: Insights From Eight Colorectal Cancer Cell Lines
Source: Food Sci Nutr. 2024 Dec 31;13(1):e4715. doi: 10.1002/fsn3.4715 (PMC11717013; doi:10.1002/fsn3.4715)
Supplement: Supplementary file 2 — Table S1. Phytochemical profiling of A. herba‐alba methanolic extract. [file FSN3-13-e4715-s002.docx]

Table S1: Phytochemical profiling of *A. herba-alba* methanolic extract

| SN^*^ | Ret. Time | Area | Name | % Area | Type | Class | Class % |
| --- | --- | --- | --- | --- | --- | --- | --- |
| 1 | 5.54 | 43551032 | Ephedrine | 0.29 | Alkaloid | Alkaloid | 0.29 |
| 2 | 13.97 | 322142390 | L-Proline | 2.16 | Amino acid | Amino acid and Derivatives | 3.56 |
| 3 | 9.75 | 118900565 | L-Valine | 0.80 | Amino acid |  |  |
| 4 | 6.94 | 20160069 | L-Alanine | 0.14 | Amino acid |  |  |
| 5 | 18.61 | 68720435 | L-5-Oxoproline | 0.46 | Amino acid derivative |  |  |
| 6 | 13.08 | 2426378 | Benzoic Acid | 0.02 | Aromatic acid | Aromatic compounds | 0.02 |
| 7 | 32.27 | 494631765 | α-Linolenic acid | 3.32 | Fatty acid | Long-chain Fats | 7.39 |
| 8 | 7.99 | 30850968 | 3,3-Dimethylacrylic acid | 0.21 | Fatty acid - saturated |  |  |
| 9 | 41.80 | 16589528 | Lignoceric acid | 0.11 | Fatty acid - Saturated |  |  |
| 10 | 44.41 | 3620267 | Hexacosanoic acid | 0.02 | Fatty acid - Saturated |  |  |
| 11 | 35.67 | 224101861 | (Z)-Docosa-8,14-diyn-1,22-diol | 1.51 | Fatty alcohol |  |  |
| 12 | 31.16 | 116744839 | 1-Octadecanol | 0.78 | Fatty alcohol |  |  |
| 13 | 40.57 | 68093975 | 1-Tetracosanol | 0.46 | Fatty alcohol |  |  |
| 14 | 31.45 | 54979038 | Phytol | 0.37 | Fatty alcohol (Diterpene) |  |  |
| 15 | 38.25 | 21739826 | 1-Monopalmitin | 0.15 | Fatty ester |  |  |
| 16 | 41.01 | 20853222 | Glycerol monostearate | 0.14 | Fatty ester |  |  |
| 17 | 40.75 | 5992260 | 1-Linolenoylglycerol | 0.04 | Fatty ester |  |  |
| 18 | 25.22 | 33482383 | Neophytadiene | 0.22 | Fatty hydrocarbon (Diterpene) |  |  |
| 19 | 42.69 | 8737859 | Hexatriacontane | 0.06 | Fatty hydrocarbon (long chain n-alkane) |  |  |
| 20 | 41.61 | 4866266 | 6-Hydroxyflavone-β-D-glucoside | 0.03 | Flavonoid glycoside | Flavonoid glycoside | 0.03 |
| 21 | 15.84 | 108981401 | 2-Methyl-Propane | 0.73 | HC short-chain alkane | HC short-chain alkane | 1.28 |
| 22 | 30.16 | 20454667 | 3-Methylene-butane | 0.14 | HC short-chain alkane |  |  |
| 23 | 12.47 | 60650910 | 3,4-Dimethyl-pent-2-ene | 0.41 | HC short-chain alkene |  |  |
| 24 | 7.23 | 124776190 | 5,5-Dimethyl-2(5H)-Furanone | 0.84 | Heterocyclic furan | Heterocycles | 2.00 |
| 25 | 11.00 | 87928835 | 2-Pyrrolidinone | 0.59 | Heterocyclic pyrrolidine |  |  |
| 26 | 8.88 | 85505456 | 5-ethenyldihydro-5-methyl-2(3H)-Furanone | 0.57 | Heterocyclic γ-lactone |  |  |
| 27 | 15.24 | 195223220 | Hexanedioic acid | 1.31 | Org. acid | Organic acids | 15.39 |
| 28 | 15.13 | 110951051 | (E)-2-Butenedioic acid | 0.75 | Org. acid |  |  |
| 29 | 14.40 | 102848139 | Butanedioic acid | 0.69 | Org. acid |  |  |
| 30 | 12.01 | 76892135 | Propanedioic acid | 0.52 | Org. acid |  |  |
| 31 | 25.17 | 60198878 | Tiglic acid | 0.40 | Org. acid |  |  |
| 32 | 9.58 | 12051284 | 2-Propenoic acid | 0.08 | Org. acid |  |  |
| 33 | 10.41 | 6281517 | (E)-2-Hexenoic acid | 0.04 | Org. acid |  |  |
| 34 | 25.55 | 1003373762 | Quinolinic acid | 6.74 | Org. acid (Hydroxy acid) |  |  |
| 35 | 17.94 | 395073065 | Malic acid | 2.65 | Org. acid (Hydroxy acid) |  |  |
| 36 | 12.11 | 154444943 | 3-Hydroxyisovaleric acid | 1.04 | Org. acid (Hydroxy acid) |  |  |
| 37 | 9.02 | 69787186 | Lactic Acid | 0.47 | Org. acid (Hydroxy acid) |  |  |
| 38 | 9.42 | 59884929 | Glycolic acid | 0.40 | Org. acid (Hydroxy acid) |  |  |
| 39 | 8.95 | 33697588 | α-Hydroxyisobutyric acid | 0.23 | Org. acid (Hydroxy acid) |  |  |
| 40 | 10.77 | 10456145 | Hydracrylic acid | 0.07 | Org. acid (Hydroxy acid) |  |  |
| 41 | 16.78 | 167947216 | 2-Methyl-3-buten-2-ol | 1.13 | Org. alcohol | Organic alcohols | 1.19 |
| 42 | 22.40 | 8956826 | Pinacol | 0.06 | Org. alcohol |  |  |
| 43 | 6.76 | 1149497 | Methyl glycolate | 0.01 | Org. hydroxy ester | Organic esters | 0.01 |
| 44 | 20.88 | 206659355 | 4-Hydroxybenzoic acid | 1.39 | Phenolic | Phenolics | 1.43 |
| 45 | 43.20 | 6832808 | 5-Methoxy-Benzoic acid | 0.05 | Phenolic acid |  |  |
| 46 | 49.59 | 10659188 | (3β)-9,19-Cyclolanost-24-en-3-ol | 0.07 | Sterol | Sterols | 0.79 |
| 47 | 50.54 | 1685651 | (3β)-9,19-Cyclolanostan-3-ol, 24-methylene-, acetate | 0.01 | Sterol acetate |  |  |
| 48 | 48.49 | 65871889 | (3β,24S)-stigmast-5-en-3-ol | 0.44 | Sterol:Phytosterol |  |  |
| 49 | 47.59 | 39108491 | Stigmasterol | 0.26 | Sterol:Phytosterol |  |  |
| 50 | 16.20 | 308659205 | 2-Deoxyribose | 2.07 | Sugar (deoxy) | Sugars and Derivatives | 55.69 |
| 51 | 19.17 | 158145119 | L-(-)-Fucose | 1.06 | Sugar (deoxy) |  |  |
| 52 | 45.85 | 62336714 | Methyl α-D-glucofuranoside | 0.42 | Sugar (methyl glycoside) |  |  |
| 53 | 29.44 | 127980744 | Galacturonic acid | 0.86 | Sugar acid |  |  |
| 54 | 26.53 | 1279351324 | Myo-Inositol (merged) | 8.59 | Sugar alcohol |  |  |
| 55 | 13.57 | 1271616839 | Glycerol | 8.54 | Sugar alcohol |  |  |
| 56 | 24.98 | 896580236 | D-Pinitol | 6.02 | Sugar alcohol |  |  |
| 57 | 22.20 | 442294144 | Xylitol | 2.97 | Sugar alcohol |  |  |
| 58 | 28.89 | 378178697 | D-Sorbitol | 2.54 | Sugar alcohol |  |  |
| 59 | 18.27 | 148543266 | L-Threitol | 1.00 | Sugar alcohol |  |  |
| 60 | 28.75 | 108554941 | Scyllo-Inositol | 0.73 | Sugar alcohol |  |  |
| 61 | 23.55 | 72033809 | D-Fucitol | 0.48 | Sugar alcohol |  |  |
| 62 | 46.17 | 4277399 | Arabinitol | 0.03 | Sugar alcohol |  |  |
| 63 | 33.85 | 476043469 | Glyceryl-glycoside | 3.20 | Sugar glycoside |  |  |
| 64 | 32.56 | 101099414 | Methyl 6-deoxy-(R,R,R,S,S)- alpha.-L-Galactopyranoside | 0.68 | Sugar glycoside |  |  |
| 65 | 37.42 | 38025333 | Methyl galactoside | 0.26 | Sugar glycoside |  |  |
| 66 | 38.93 | 1086978010 | Sucrose | 7.30 | Sugar: Disaccharide |  |  |
| 67 | 36.93 | 136530620 | D-(+)-Cellobiose (merged) | 0.92 | Sugar: Disaccharide |  |  |
| 68 | 40.32 | 62664198 | D-(+)-Trehalose | 0.42 | Sugar: Disaccharide |  |  |
| 69 | 41.28 | 60770941 | 2-α-Mannobiose (merged) | 0.41 | Sugar: Disaccharide |  |  |
| 70 | 31.02 | 43762270 | Melibiose (merged) | 0.29 | Sugar: Disaccharide |  |  |
| 71 | 39.21 | 26226295 | 3-α-Mannobiose | 0.18 | Sugar: Disaccharide |  |  |
| 72 | 43.79 | 15687484 | D-(+)-Turanose | 0.11 | Sugar: Disaccharide |  |  |
| 73 | 44.90 | 8087728 | D-Lactose | 0.05 | Sugar: Disaccharide |  |  |
| 74 | 42.08 | 2364796 | Maltose | 0.02 | Sugar: Disaccharide |  |  |
| 75 | 24.41 | 280214810 | D-(-)-Fructofuranose | 1.88 | Sugar: Monosaccharide |  |  |
| 76 | 26.16 | 214678790 | β-D-Galactofuranose | 1.44 | Sugar: Monosaccharide |  |  |
| 77 | 27.98 | 173645103 | β-D-Allopyranose (merged) | 1.17 | Sugar: Monosaccharide |  |  |
| 78 | 27.76 | 142948965 | α-L-Mannofuranose | 0.96 | Sugar: Monosaccharide |  |  |
| 79 | 31.73 | 70170796 | α-D-Mannopyranose | 0.47 | Sugar: Monosaccharide |  |  |
| 80 | 31.67 | 58015521 | D-Xylose | 0.39 | Sugar: Monosaccharide |  |  |
| 81 | 32.48 | 32790174 | Glucose | 0.22 | Sugar: Monosaccharide |  |  |
| 82 | 40.80 | 1697414 | (R,S,R,R,S)-β-L-(-)-Fucopyranose | 0.01 | Sugar: Monosaccharide |  |  |
| 83 | 12.60 | 674949614 | exo-Borneol | 4.53 | Terpene: Monoterpene alcohol | Terpenes: Mono- and Sesqui-terpenes | 10.94 |
| 84 | 8.57 | 219583781 | Eucalyptol | 1.47 | Terpene: Monoterpene alcohol |  |  |
| 85 | 23.51 | 63131500 | Linalool | 0.42 | Terpene: Monoterpene alcohol |  |  |
| 86 | 11.90 | 44317829 | Terpinen-4-ol | 0.30 | Terpene: Monoterpene alcohol |  |  |
| 87 | 11.18 | 391513487 | Camphor (merged) | 2.63 | Terpene: Monoterpene ketone |  |  |
| 88 | 15.90 | 132754197 | (Z)-Linolool oxide | 0.89 | Terpene: Monoterpene oxide |  |  |
| 89 | 8.13 | 1540933 | 8,9-Dehydrothymol methyl ether | 0.01 | Terpene: Monoterpene Phenol |  |  |
| 90 | 40.62 | 70344568 | Thymol-β-d-glucopyranoside | 0.47 | Terpene: Monoterpene phenol glycoside |  |  |
| 91 | 19.60 | 29993598 | β-Eudesmol | 0.20 | Terpene: Sesquiterpene alcohol |  |  |

^*^ Compounds were ordered based first on their “Type”, then, “% Area” from highest to lowest.
